# Pressure-induced dissociation of water molecules in ice VII

**DOI:** 10.1038/srep12551

**Published:** 2015-07-27

**Authors:** Toshiaki Iitaka, Hiroshi Fukui, Zhi Li, Nozomu Hiraoka, Tetsuo Irifune

**Affiliations:** 1 Computational Astrophysics Laboratory, RIKEN, 2-1 Hirosawa, Wako, Saitama 351-0198, Japan; 2 Center for Novel Material Science under Multi-Extreme Conditions, Graduate School of Material Science, University of Hyogo, Kamigori, Hyogo 678-1297, Japan; 3 School of Materials Science and Engineering, Hefei University of Technology, Hefei, Anhui 230009, China; 4 National Synchrotron Radiation Research Center, Hsinchu 30076, Taiwan; 5 Geodynamics Research Center, Ehime University, 2-5 Bunkyo-cho, Matsuyama, Ehime 790-8577, Japan; 6 Earth-Life Science Institute, Tokyo Institute of Technology, Tokyo 152-8550, Japan

## Abstract

The neutron diffraction pattern of D_2_O ice was recently measured at pressures up to 52 GPa by Guthrie *et al.*, who proposed an octahedral interstitial model for ice at pressures above 13 GPa to account for the deviation of the observed crystal structure from that of ice VII. In this article, the octahedral interstitial model was re-examined in terms of the interstitial occupancy and X-ray Raman spectroscopy (XRS) spectra. The interstitial occupancy calculated using first-principles molecular dynamics simulations was negligibly small compared to that of the interstitial model. The oxygen *K-*edge spectra calculated for the interstitial model exhibited two additional low-energy peaks originating from water molecules and hydroxides that are interacting with interstitial protons, respectively, whereas these low-energy peaks were not observed in the experimentally measured spectra. These results suggest that the interstitial model cannot explain the XRS spectra of ice VII at pressures above 13 GPa and that more precise structure measurements and analyses are necessary to reveal the nature of the pressure-induced transition.

Water is a simple molecule consisting of two hydrogen atoms and one oxygen atom. However, water exhibits an extremely complex phase diagram that includes more than fifteen phases. Under compression at room temperature, water transforms at 2.2 GPa from ice VI to ice VII, which consists of two interpenetrating sublattices of water molecules forming a bcc oxygen lattice and two random hydrogen-bond networks. As the pressure is increased from 2.2 GPa to 60 GPa, the bcc oxygen lattice remains stable, with reduced lattice constants; however, the hydrogen-bond networks evolve from statically random to dynamically random and eventually to the symmetric state. In this process, ice VII exhibits intricate phenomena originating from the complex proton dynamics, and X-ray diffraction provides very little information about these phenomena.

Although ice VII has been traditionally considered as a single stable phase of water ice, the literature contains several reports on the transition of the structure and properties at approximately 10–20 GPa^1^. The transition of the electric charge carrier at approximately 10 GPa was observed by impedance spectroscopy^2^. The maximum dissociation ratio of water molecules irradiated by X-rays was observed^3^ using X-ray Raman spectroscopy (XRS)^4^,^5^. The deviation of the neutron diffraction pattern of deuterated ice at pressures above *P*_*c*_ = 13 GPa from that of the ice VII structure was reported by Guthrie *et al.*^6^, who proposed an octahedral interstitial model with substantial deuteron occupancy (~one deuteron/(unit cell)) at the octahedral interstitial voids of the oxygen lattice. Guthrie *et al.* concluded that some D_2_O molecules are dissociated at pressures above *P*_*c*_, and that protonic species begin to localise at the interstitial sites. A new low-frequency Raman band in the metastable region of ice VII has been reported^7^ to appear at 14 GPa and at 80 K and 10 K but not at 140 K, suggesting^8^ the existence of a solid-solid critical point (*T*_*c *_≈ 100 K, *P*_*c*_ ≈ 14 GPa) as a possible common origin of these anomalies.

Therefore, scrutinising the nature of the transition at *P*_*c*_, especially the change of the hydrogen position predicted by the octahedral interstitial model^6^, would be interesting. In this article, the octahedral interstitial model was re-examined in terms of the interstitial occupancy and the XRS spectra. The interstitial occupancy of ice VII was calculated using a first-principles molecular dynamics simulation, and the results were compared to those predicted by the interstitial model. In addition, because some of the water molecules in ice VII should be dissociated to supply protonic species at the octahedral interstitial sites, additional XRS spectral peaks^9^ should be observed at energies that differ from that of ice VII. Following this idea, the theoretical oxygen *K*-edge XAS spectrum, which can be directly compared to the XRS spectrum measured in the dipole limit, was calculated for the ice VII model and the octahedral interstitial model; these spectra were subsequently compared with the experimental XRS spectra.

## Theoretical and Experimental

**Figure 1:**
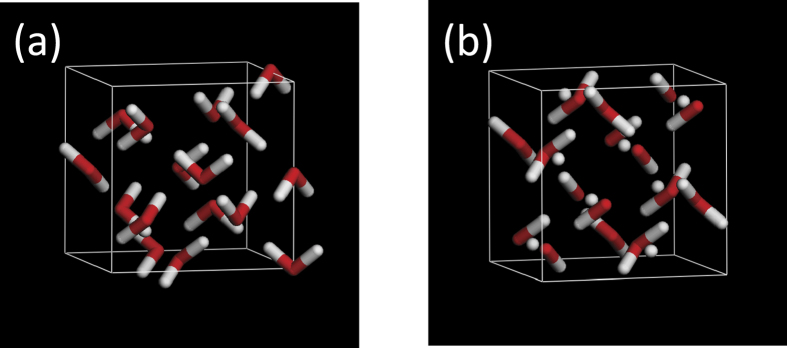
Unit cell of (**a**) the ice VII model and (**b**) the octahedral interstitial model.

First, the occupancy of interstitial hydrogen was evaluated using the trajectories generated by first-principles molecular dynamics simulations. The initial structure was the geometrically optimised ice VII model (Fig. 1(a)) containing 16 water molecules in a cubic unit cell and the experimental lattice constant^10^,*a*_0_, at pressures between 2 GPa and 40 GPa. The molecular dynamics simulations were performed for 100 ps with a time step of 0.1 fs using an NVT ensemble at temperatures of 300 K and 800 K controlled by a Nose-Hoover thermostat^11^. The occupancy was evaluated for each snapshot by counting the number of protons within the sphere of radius *a*_0_/8 at the centre of the octahedral interstitial sites. The first-principles molecular dynamics simulations were performed using the Vienna *ab initio* simulation package (VASP) code^12^. Electronic structure calculations were performed with the projector augmented wave potentials^13^ in the generalised gradient approximation GGA-PW91^14^. The electron orbitals were represented by plane waves with an energy cutoff of 700 eV and the Gamma k-point.

Then, the oxygen *K*-edge XAS spectra were calculated using the 2 × 2 × 2 supercell of the ice VII model (Fig. 1(a)) as well as that of the interstitial model with half of water molecules dissociated into OH^−^ at the bcc-site and H^+^ at the octahedral site (Fig. 1(b)). Both models consist of 16 water molecules in the unit cell and have no total electric dipole moment. The evaluation of the XAS spectra was based on density functional theory as implemented in the WIEN2k code^15^. The calculations were performed with the GGA-PBE^16^ exchange-correlation functional, periodic boundary conditions with Brillouin zone integration with 3 × 3 × 3 Monkhorst-Pack k-points, and *R*_*mt*_*K*_max_ = 3, where *R*_*mt*_ is the smallest atomic sphere radius and *K*_max_ is the magnitude of the largest *K* vector in the expansion of the Kohn–Sham equation. These parameters are sufficient for systems with hydrogen atoms. The convergence was tested for selected calculations by increasing the parameters to *R*_*mt*_*K*_max_ = 5 and 5 × 5 × 5 Monkhorst-Pack k-points. The XAS spectra were obtained using the Fermi golden rule with transition moments between a core electron and valence and conduction band states^17^ with the spectrometer broadening S = 1.4 eV and the lifetime broadening of the core excited state GAMMA0 = 0.18 eV^18^. The electron relaxation and final state interaction between the excited electron and the core hole were modelled by removing one 1*s* core electron and placing it in the valence band (i.e., a full core-hole approximation).

Finally, XRS spectra of the oxygen *K-*edge for dense ice were measured at the BL12XU beamline of SPring-8, Japan. X-rays with an energy of 9.887 keV were incident on the target loaded into a Be gasket; the X-rays scattered at an angle of 30° were counted using the backscattering analyser operated at the Si (5 5 5) reflection. Nano-polycrystalline diamonds^19^ with a culet size of 0.4 mm were used as anvils to obtain a sample with a relatively large volume under high-pressure conditions. The initial diameter of the sample chamber was 0.2 mm. The XRS spectrum was compared with the theoretical XRS spectrum, which is well approximated by the XAS spectrum when the momentum transfer *Q* is small (*Q·r* ≪ 1) with respect to the radius *r* of the core wave function. This condition is satisfied in the present measurement where *Q* = 2.7 *Å*^*−*1^ and *r* = 0.069 *Å*. XRS is more frequently used in high-pressure experiments for light elements than XAS because XRS uses hard X-rays that can easily penetrate the sample cell.

## Results and Discussion

**Figure 2:**
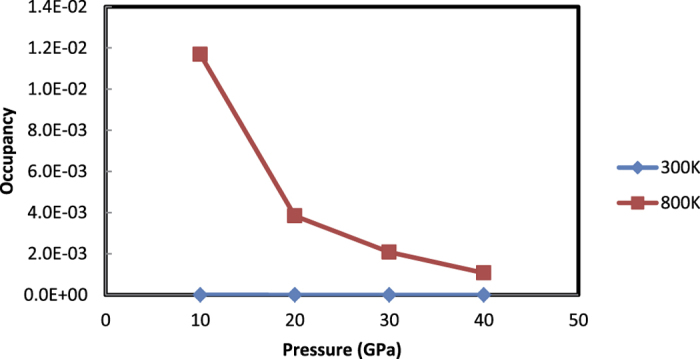
Interstitial occupancy as a function of pressure evaluated by the first principles molecular dynamics simulation.

First, the occupancy of the interstitial hydrogen evaluated by the molecular dynamics simulation is shown in Fig. 2. The occupancy of the interstitial proton was less than 10^−4^ at 300 K and less than 2 × 10^−2^ even at an elevated temperature of 800 K, implying that no significant water dissociation occurs in the ice in this pressure and temperature range. This result is in contrast to the occupancy of approximately one deuteron/(unit cell) predicted by the interstitial model. Moreover, it was found that the interstitial model is more unstable by 2.0 eV per water molecule in energy compared to the ice VII model.

**Figure 3:**
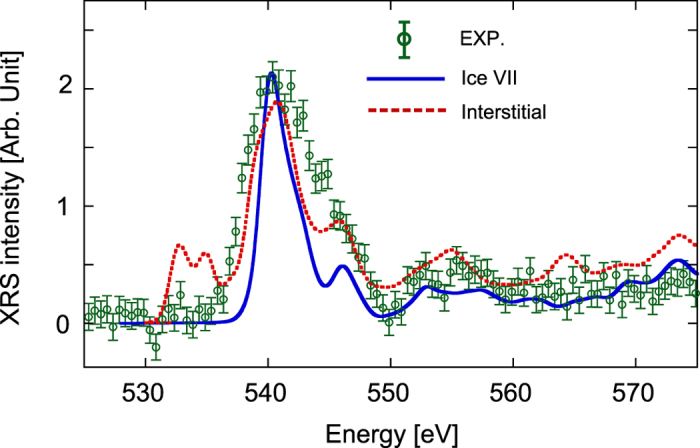
Oxygen *K*-edge XRS spectrum of dense ice. Green circles are the XRS data measured at 53 GPa with energy resolution of 1.4 eV. The error bars are statistical one, i.e., square root of the intensity. Blue solid line and red broken line are the theoretical spectra calculated at 50 GPa for the ice VII model and the octahedral interstitial model, respectively. The main peak of each theoretical spectrum is aligned to the experimental main peak for comparison.

Second, to study the effect of water dissociation, the oxygen *K*-edge XAS spectra averaged over all oxygen atoms (Fig. 3) were calculated using the 2 × 2 × 2 supercell of the normal ice VII model (Fig. 1a) and that of the interstitial model (Fig. 1b). The ice VII model exhibits a typical spectrum of ice VII with the main peak, whereas the interstitial model exhibits two additional low-energy peaks. In order to reveal the origin of these low-energy peaks, the density of states of the interstitial model was calculated and compared with that of the ice VII model (Supplementary Fig. S1). Two unoccupied bands appeared in the band gap, corresponding to the two peaks in the XRS spectrum of the interstitial model. The real-space density of these bands was plotted in Supplementary Fig. S2, from which it is interpreted that the band in the energy range between 0 eV and 4 eV is the unoccupied states due to the interaction between the water molecule and the dissociated proton; the band between 4 eV and 7 eV is the unoccupied states due to the interaction between the OH^−^ anion and the dissociated proton.

Finally, the oxygen *K-*edge XRS spectrum (Supplementary Fig. S3) was measured at 53 GPa with energy resolution of 1.4 eV using Double Crystal Monochromator (DCM). The background of the spectrum mostly consists of Compton scattering from the high pressure apparatus and also from the sample itself. It has a maximum around several tens of electron volts in energy loss and a long tail toward the larger energy loss side. The tail was fitted to a function of *y* = *A*/(*x* − *B*) + *C* where A = 10000, B = 490 eV and C = 1750 (Supplementary Fig. S3). The experimental spectrum corrected by subtracting the background (Fig. 3) is compared with the theoretical spectra of the ice VII model and the interstitial model. The oxygen *K-*edge XRS spectra were also collected under various pressures greater than 40 GPa; the features of the resulting spectra were similar to that obtained at 53 GPa. It should be noted that absence of the peaks corresponding to the dissociated species is inconsistent with the interstitial model. The present result is, however, consistent with the report by Fukui *et al.*^3^ that the peak due to X-ray-induced water dissociation^20^, which is expected to appear in the same energy region, was not observed at pressures above 37 GPa.

A distinct pre-edge peak at 534.6 eV, which has been reported for ice VII at 2.2 GPa^21^ and other lower-pressure phases^21^,^22^,^23^,^24^,^25^, was not observed in this measurement, probably due to the insufficient energy resolution of the measurement and the weaker intensity of the peak compared to the low-pressure data. It has been argued^24^,^25^ that the pre-edge peak will disappear when hydrogen bonds are symmetrized and ice X is formed. Therefore the XRS spectra of ice VII at pressures from 2 GPa to 50 GPa were calculated with high resolution (0.3 eV) and shown in Supplementary Fig. S4 where the pre-edge is indicated by a solid arrow. The intensity of the pre-edge peak becomes weaker as the pressure increases. At 50 GPa, the pre-edge peak still exists but becomes very weak. With low resolution (1.4 eV), the pre-edge peak was absorbed into the main peak and could not be resolved at all pressures. The oxygen *K-*edge XRS spectrum with high energy resolution (Supplementary Fig. S3) was also measured at 50 GPa with using a High-Resolution Monochromator in addition to DCM. The statistics of the data was not sufficient to identify the weak pre-edge peak. High resolution XRS measurements with high statistics are demanded to study this pre-edge feature.

In summary, the deviation in the neutron diffraction pattern at pressures above 13 GPa^6^ from that of ice VII was re-examined. The deviation may indicate a change in the crystal structure of ice VII; however, the octahedral interstitial model proposed by Guthrie *et al.*^6^ appears to be inconsistent with the interstitial occupancy obtained by the first-principles molecular dynamics simulation and with the experimental XRS spectra. To identify the structural change observed at pressures above 13 GPa, careful structural analysis based on high-quality neutron diffraction data under high pressures^26^,^27^,^28^ is indispensable.

## Additional Information

**How to cite this article**: Iitaka, T. *et al.* Pressure-induced dissociation of water molecules in ice VII. *Sci. Rep.* **5**, 12551; doi: 10.1038/srep12551 (2015).

## Supplementary Material

Supplementary Information
