# Supplementary material for: Pressure-induced dissociation of water molecules in ice VII
Source: Sci Rep. 2015 Jul 27;5:12551. doi: 10.1038/srep12551 (PMC4515761; doi:10.1038/srep12551)
Supplement: Supplementary Information [file srep12551-s1.pdf]

Pressure-induced dissociation of water molecules in ice VII

Toshiaki Iitaka[1]*, Hiroshi Fukui[2], Zhi Li[3,1], Nozomu Hiraoka[4], Tetsuo Irifune[5,6]

[1]Computational Astrophysics Laboratory, RIKEN, 2-1 Hirosawa, Wako, Saitama 351-0198, Japan

[2]Center for Novel Material Science under Multi-Extreme Conditions, Graduate School of Material Science, University of Hyogo, Kamigori, Hyogo 678-1297, Japan

[3]School of Materials Science and Engineering, Hefei University of Technology, Hefei 230009, Anhui, China

[4]National Synchrotron Radiation Research Center, Hsinchu 30076, Taiwan

[5]Geodynamics Research Center, Ehime University, 2-5 Bunkyo-cho, Matsuyama, Ehime 790-8577, Japan

[6] Earth-Life Science Institute, Tokyo Institute of Technology, Tokyo 152-8550, Japan

April 21, 2015

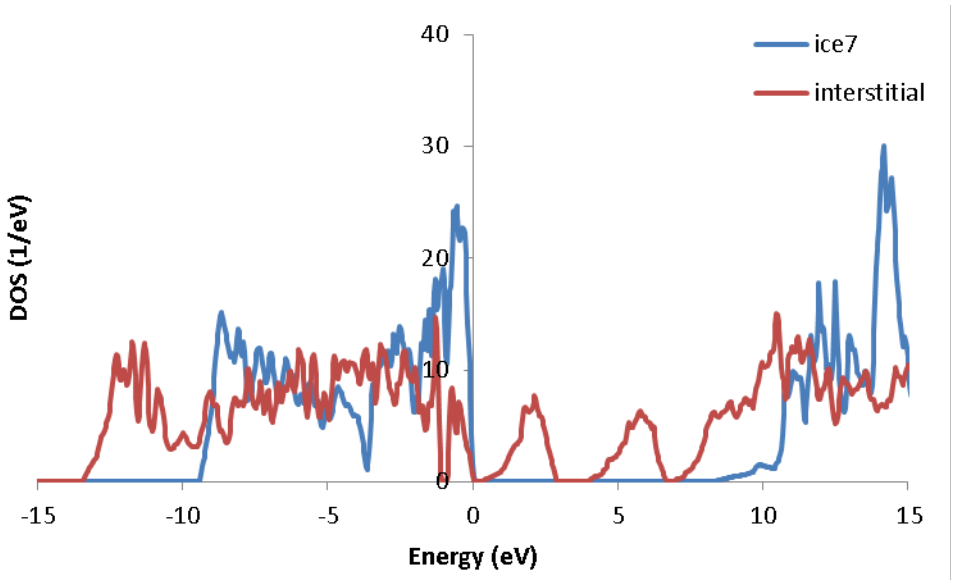

Supplementary Figure S1 | Density of states of the interstitial model and the ice VII model calculated with 1 × 1 × 1 unit cell and 6× 6 × 6 k-points.

(a)

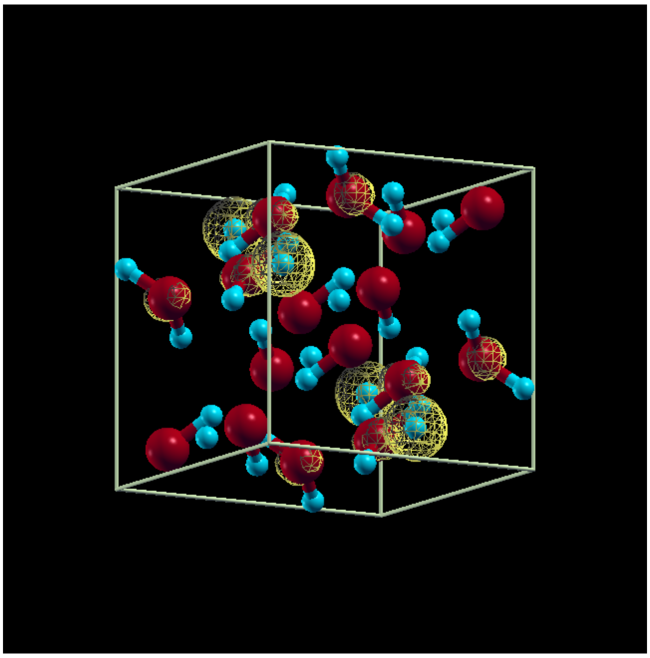

(b)

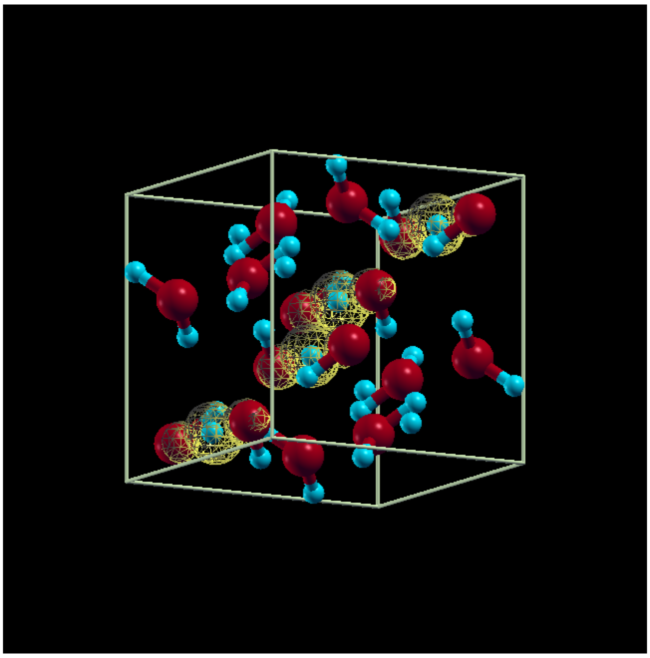

Supplementary Figure S2 | Density distribution of the unoccupied bands of the interstitial model in the energy range (a) between 0 eV and 4 eV and (b) between 4 eV and 7 eV.

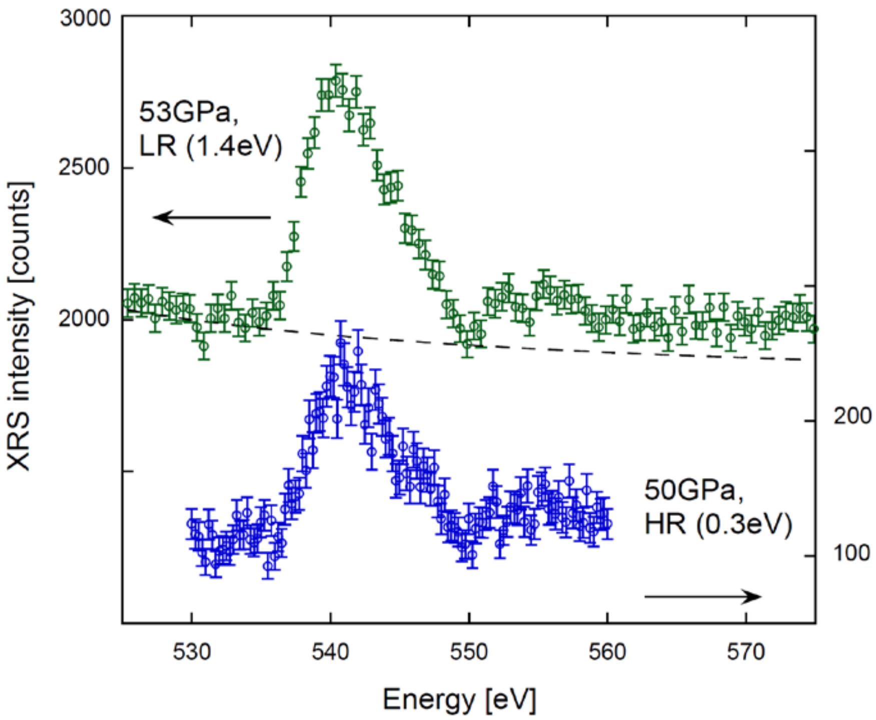

Supplementary Figure S3 | Experimental XRS spectra with energy resolutions 0.3 eV and 1.4 eV. The background was fitted to a function of $y = A/(x-B)+C$ where A=10000, B=490 eV and C=1750

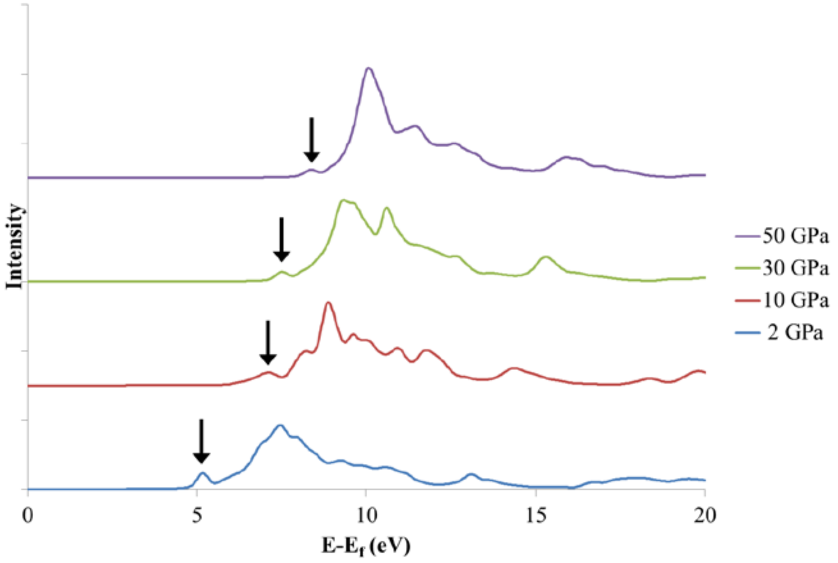

Supplementary Figure S4 | XRS spectra of ice VII at pressures from 2 GPa to 50 GPa with high resolution (0.3 eV). The pre-edge is indicated by a solid arrow.
